# Mycobiome Diversity in Traditionally Prepared Starters for Alcoholic Beverages in India by High-Throughput Sequencing Method

**DOI:** 10.3389/fmicb.2019.00348

**Published:** 2019-03-05

**Authors:** Shankar Prasad Sha, Mangesh Vasant Suryavanshi, Jyoti Prakash Tamang

**Affiliations:** DAICENTRE (DBT-AIST International Centre for Translational and Environmental Research) and Bioinformatics Centre, Department of Microbiology, School of Life Sciences, Sikkim University, Gangtok, India

**Keywords:** dry starter, mycobiome, yeasts, molds, high-throughput sequencing

## Abstract

*Chowan, dawdim, humao, hamei, khekhrii*, and *phut* are sun-dried starters used for preparation of alcoholic beverages in North East regions of India. We attempted to profile the mycobiome community in these starters by high-throughput sequencing (HTS) method. All fungal populations were found to be restricted to Ascomycota (67–99%), Zygomycota (0.7–29%), Basidiomycota (0.03–7%), and Chytridiomycota (0.0003%). We found 45 core operational taxonomic units (OTUs) which were universally present and were further weighed to 41 genera level and 22 species level taxonomy. A total number of 594 fungal species were detected by HTS including common species (224), unique species (133) and rare-species (237) in samples of starters. Unique species were recorded in *phut* (40 species), *khekhrii* (28), *hamei* (23), *dawdim* (21), *chowan* (13), and *humao* (8), respectively. Most of the fungal families were found to correlate to a type of nutritional mode and growth morphologies of the community, where saprotrophic mode of mold species were more dominant, whereas morphotypes were more dominant in yeast species.

## Introduction

Traditionally prepared sun-dried cereal-based amylolytic/alcoholic starters, in the form of round/oval/flattened balls of varied sizes for production of mild-alcoholic beverages, are common in South East Asia (Hesseltine, 1983; Steinkraus, 1996; Nout and Aidoo, 2002; Tamang, 2010a). Usually three types of mixed cultures are traditionally used as starters to convert cereal starch to sugar and then to alcohol and organic acids (Hesseltine et al., 1988; Tamang and Fleet, 2009; Tamang, 2010a,b). These are (1): dried starter consisting of consortia of amylase/alcohol producing-yeasts, filamentous molds and bacteria, which are locally called *marcha* in India, Nepal and Bhutan (Tsuyoshi et al., 2005), *chiu/chu/daque* in China (Chen et al., 2014; Xu et al., 2017), *nuruk* in Korea (Jung et al., 2012), *ragi* in Indonesia (Surono, 2016), *loog-pang* in Thailand (Limtong et al., 2002), *benh men* in Vietnam (Dung et al., 2007) and *dombea* in Cambodia (Ly et al., 2018); (2): mixed culture of molds *Aspergillus oryzae* and *A. sojae* in the form of a starter called *koji* in Japan for making *saké*, distilled liquor, and several fermented soybean products such as *miso* and *shoyu* (Kitamura et al., 2016), and (3): large compact cakes made up of whole-wheat flour with yeasts and filamentous molds to ferment starchy substrates for production of alcohol, mostly in China (Tamang, 2010a). Microbiota associated with traditionally prepared Asian dried starters are starch-degrading genera of molds *Actinomucor, Amylomyces, Aspergillus, Mucor, Neurospora, Penicillium, Rhizopus* (Hesseltine et al., 1988; Tamang et al., 1988; Nikkuni et al., 1996; Nout and Aidoo, 2002; Chen et al., 2014; Tamang et al., 2016a); amylolytic and alcohol-producing yeasts genera mostly *Candida, Debaryomyces, Dekkera*, *Galactomyces, Geotrichum, Hansenula, Hanseniaspora, Issatchenkia*, *Kazachstania*, *Kluyveromyces, Pichia, Saccharomyces, Saccharomycodes, Saccharomycopsis, Schizosaccharomyces, Torulaspora*, *Torulopsis, Wickerhamomyces*, and *Zygosaccharomyces* (Hesseltine and Kurtzman, 1990; Tamang and Sarkar, 1995; Tsuyoshi et al., 2005; Jeyaram et al., 2008; Lv et al., 2012, 2013; Chakrabarty et al., 2014; Sha et al., 2016, 2017, 2018) and few genera of bacteria, mostly *Pediococcus, Lactobacillus* (Hesseltine and Ray, 1988; Tamang and Sarkar, 1995; Sujaya et al., 2001; Tamang et al., 2007; Chakrabarty et al., 2014).

Since the culture-dependent method can only isolate the culturable microorganisms from samples using media, the culture-independent method may profile all microbial communities, including both those that are culturable and unculturable in food samples, by extracting the whole genomic DNA directly from small amount of samples (Roh et al., 2010; Jung et al., 2012; Puerari et al., 2015; Sha et al., 2018). Culture-independent methods, including pyrosequencing and high-throughput amplicon sequencing, are commonly applied for profiling microbiome of natural food fermentation within a short time and with more accuracy (Alegría et al., 2011; Cocolin et al., 2013; Chen et al., 2014; Mayo et al., 2014; Puerari et al., 2015; Tamang et al., 2016b; Shangpliang et al., 2018). Application of the amplicon-based high-throughput sequencing has been demonstrated for the monitoring of microbial populations between different strains within a species (Ercolini et al., 2012), and inter- and intra-species diversity within a particular genus or among genera (Yan et al., 2013).

**FIGURE 1:**
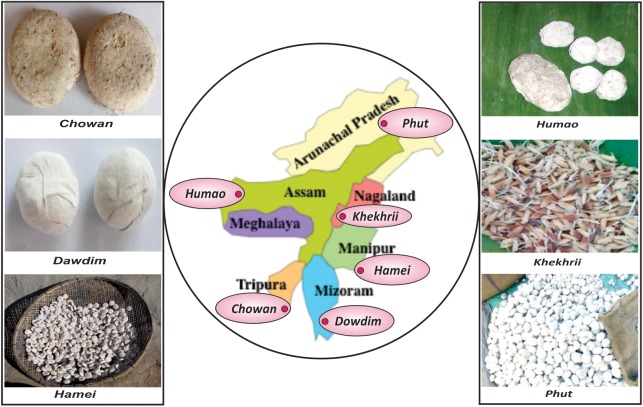
Origin and types of sun-dried alcoholic starter cultures in eight North East states of India.

Drinking of traditional alcoholic beverages and drinks is the distinct dietary culture and practices of ethnic people of North East India^1^ with strong ritualistic and ethnical importance (Tamang, 2010a; Tamang et al., 2016a). Traditionally prepared sun-dried starters such as *dawdim, hamei, humao, khekhrii, chowan*, *phut*, etc., in North East states of India (Anupma et al., 2018) are commonly used by diverse groups of ethnic people to prepare mild-alcoholic (4–5%) beverages with sweet taste, providing a high source of calories and minerals (Thapa and Tamang, 2004, 2006; Tamang and Thapa, 2006; Tamang et al., 2012). In this study we selected six different starters, such as *chowan* of Tripura, *dawdim* of Mizoram, *hamei* of Manipur, *humao* of Assam, *khekhrii* of Nagaland and *phut* of Arunachal Pradesh, from North East states of India (Figure 1). All these amylolytic/alcoholic starters are dry, hard, with different shapes of round to flattened solid ball like structure, sizes ranging from 1.2 to 11.2 cm in diameter, and all creamy to dusty white in color. Except for *khekhrii*, all other starters are traditionally prepared from soaked rice/wheat, mixed with some locally available wild plants, added with previously prepared powdered starters (1–2%), and kneaded into round to flattened cakes by adding water. The mixtures are covered with fern fronds/paddy straws/jute sags, fermented for 1–3 days at room temperature; and finally sun dried (2–3 days) to get dry starters, which can be kept for a year or more (Tamang et al., 2016a; Anupma et al., 2018). *Khekhrii* is the only amylolytic/alcoholic starter in North East India, which is prepared by fermenting germinated sprouted-rice grains and then sun-dried to use as dry starters to prepare the local alcoholic drink (Anupma et al., 2018). Sha et al. (2018) studied the fungal diversity in *chowan, dawdim, hamei, humao, khekhrii*, and *phut*, based on the culture-dependent method using ITS-PCR and a culture-independent approach by PCR-DGGE analysis. In this paper, we attempted to understand the “ethno-microbiology” of mycobiome diversity in *chowan, dawdim, hamei, humao, khekhrii*, and *phut* by using the high-throughput sequencing method supported by bioinformatics interpretation.

## Materials and Methods

### Sample Collection

Six samples of starter: *chowan* of Tripura, *dawdim* of Mizoram, *hamei* of Manipur, *humao* of Assam, *khekhrii* of Nagaland and *phut* of Arunachal Pradesh (Figure 1) were collected immediately after the preparation (fermentation and sun-drying) from different places of North East India. The average pH of these starter samples was 4.9 ± 0.2. Samples were kept in sterile containers, leveled, transported to the laboratory and stored at room temperature in a desiccator; dried starter can retain its potency *in situ* for more than a year in the moist-free condition (Tamang et al., 1988).

### Community DNA Extraction

Firstly, dried starter samples were powdered with the help of sterile mortar and pestle and 1g of powdered sample were taken and homogenized in 9 ml of 0.85% physiological saline and subsequently filtered through 4 layers of sterile cheese-cloth. The resulting filtered solutions were centrifuged at 14000 *g* for 10 min at 4°C (Lv et al., 2013; Sha et al., 2018) and then the pellets were subjected to total community DNA extraction using the ProMega DNA extraction kit (ProMega, United States) according to the manufacturer’s instructions. Subsequently, the RNA was eliminated from the cellular lysate by administering the RNase solution after incubation at 35°C for 15 min. The residual proteins were removed by adding protein precipitation solution and then centrifuging at maximum speed. Finally, the DNA was precipitated by adding isopropanol, and purified with two washes of 70% ethanol. Quality of DNA was checked on 0.8% agarose gel by measuring the concentration using Nano-Drop spectrophotometer (AG-6135, Eppendorf, Germany). The DNA was kept at -20°C until further processing.

### Sequencing of Fungal ITS2 Gene Region and Pre-processing

Internal Transcribed Spacer (ITS) 2 region of fungi was targeted for taxonomic profiling by amplification using ITS2F (GCATCGATGAAGAACGCAGC) and ITS2R (TCCTCCGCTTATTGATATGC) primers (Blaalid et al., 2013) due to its high interspecific variability which also conserved primer sites with multiple copies (Schoch et al., 2012). A composite sample for sequencing was made by combining equimolar ratios of amplicons from the samples, followed by gel purification with a QiagenMinElute gel extraction kit to remove potential contaminants and PCR artifacts. Amplicon libraries were purified by 1X AMpureXP beads, which were checked on an Agilent DNA 1000 chip on Bioanalyzer 2100, and finally quantified by Qubit Fluorometer 2.0 using Qubit dsDNA HS Assay kit (Life Technologies). MiSeqIllumina platform using 2 × 250 bp chemistry sequencing was performed. Pre-processing of downstream analysis for raw read was completed as described by Comeau et al. (2017), as follows: firstly, raw read quality from sequencer was checked for the average and range of the Phred quality scores along the reads (1 to 300 bp), for both forward and reverse reads independently, to pass it to the next steps using FastQC^2^; secondly, removal of adapter sequences through cut adapt tool (Martin, 2011); thirdly, adapter cleaned paired-end reads files merged using the PEAR (v0.9.10) program (Zhang et al., 2014) with default settings; fourthly, FASTQ stitched files were converted to FASTA and removed any sequences that had an “N” in them with run_fastq_to_fasta.pl command lin; and lastly, chimeric sequences were removed with VSEARCH tool (Rognes et al., 2016) using UNITE_uchime_ITS2only_01.01.2016.fasta reference dataset.

### Downstream Analysis of ITS Gene Region Reads

The downstream analysis of chimera free FASTA files was done for detecting the taxonomic classification and their functional guided activity. For taxonomic classification of each sequence, we performed the diversity analysis in the QIIME 1.9 environment (Caporaso et al., 2010). Sequence reads were combined in a single FASTA file with guided metadata files and further steps were done accordingly as described by Comeau et al. (2017). Fungal operational taxonomic units (OTUs) were analyzed by an open reference-based OTU picking approach using UNITE reference database as UNITE_sh_refs_qiime_ver7_dynamic_20.11.2016.fasta. The OTU picking was carried out using the sortmerna_sumaclust method with a similarity threshold of 97%. Taxonomic assignments were performed using mother classifier (Schloss et al., 2009).

We performed the analysis with PIPITS (Gweon et al., 2015) and FUNGuild environments (Nguyen et al., 2016) for functional guided activity determination. The ITS2 region was extracted with ITSx, clustered into OTUs with VSEARCH^3^ at 97% sequence similarity, and chimera removal was performed using the UNITE UCHIME reference data set. Representative sequences were assigned taxonomic classification with the RDP classifier against the UNITE fungal ITS reference data set at a confidence threshold of 0.85. We generated otu_table_funguild file by using pipits_funguild.py command line. This OTU table was used to run the online Guilds application to assign functional information to OTUs in high-throughput sequencing datasets^4^.

### Other Data Analysis

Alpha diversity analyses of the mycobiome were tested using QIIME platform and with the alpha_diversity.py script. For the continuous variables, non-parametric t-test was used, and for categorical variables between groups, either the Pearson chi-square or Fisher’s exact test was used depending on assumption validity. Data analyses were performed by statistical environment R^5^. Phylum level abundance plots, bubble plots and heatmap were derived by ggplot2 package (Wickham, 2016), core microbiome heatmap were derived by microbiome package (Lahti and Shetty, 2017) and correlation plot by corplot package (Wei and Simko, 2017). The filtered OTUs based (less than 1% abundance value) rare-phylotypes heatmap were derived by ggplot2 package (Wickham, 2016). UPGMA based dendrogram was created using the Pearson similarity coefficient. Alpha diversity indices like Chao, Shannon, and Simpson were calculated after rarefying all samples to the same sequencing depth (Cox et al., 2017). Non-metric multidimensional scaling plots (NMDS) based on Bray-Curtis distance matrix was constructed to carry out the beta-diversity analysis.

### Data Availability

The sequences obtained from high-throughput sequencing effort were submitted to NCBI, which are available under SRA accession: SRP150043 and BioProject ID:PRJNA474271.

## Results and Discussion

**FIGURE 2:**
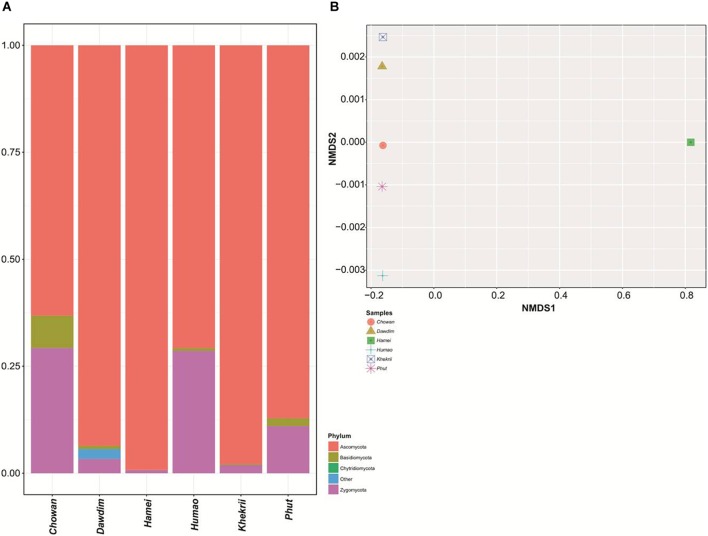
**(A)** Barplot showing the phylum level diversity and their abundance distribution found in starters. **(B)** NMDS plot showing the beta diversity clustering pattern in tested subjects.

The present study reveals the mycobiome diversity in the same samples of *chowan, dawdim*, *hamei, humao*, *khekhrii*, and *phut* by culture-independent method using high-throughput sequencing approach, which permits the analysis of hundreds of nucleotide sequences (Roh et al., 2010). We generated 5213436 paired end sequences and were clustered into operational taxonomic units (OTUs) by single linkage clustering with 97% sequence similarity. About 2488812 high quality sequences (sequence lengths: 374 ± 31 nucleotides) and normalization were done on 292996 per sample for the study, which were assembled into 6097 global and species-level OTUs. All OTUs with <2 reads in total and those not representing fungi were omitted. OTU-table was generated for further taxonomy-based analysis. Samples diversity surveillance for the fungal population was analyzed by intra-sample variations through the alpha diversity measures (Table 1). The diversity indices provide an idea about the expected diversity values, like *goods coverage* index within 0.990 to 0.998. Observed OTUs values were found to be wide and within the range of 702 to 3037. Among the six starters, *hamei* had the highest OTUs. Phylum level abundance varied in each sample and was mostly limited to taxa Ascomycota, Basidiomycota, Chytridiomycota and Zygomycota (Figure 2A). The samples were discrete on the NMDS plots using OTUs level variations (Figure 2B). All fungal populations were found to be restricted to only Ascomycota (67–99% of abundance), Zygomycota (0.7–29%) and Basidiomycota (0.03–7%), however, Chytridiomycota (0.0003%) was also enlisted in *khekhrii* sample. High prevalence of Ascomycota phylum was also reported in similar types of dry starters of Asia such as *nuruk* of Korea (Jung et al., 2012; Bal et al., 2016), and *daqua* of China (Li et al., 2011; Chen et al., 2014; Xu et al., 2017). In the present study quantitative differences were observed for the presence of fungal taxa in all six starters, which could be the consequence of differences in the traditional methods of production of starters, use of wrapping materials and varied fermentation time (Jeyaram et al., 2011; Bora et al., 2016; Anupma et al., 2018). The Alpha diversity estimation of all starters using species richness and non-parametric Shannon index showed dominance of phylum Ascomycota over the Zygomycota. A similar observation was also reported in similar types of dry starters of India: *thiat* of Meghalaya state (Sha et al., 2017), and in *marcha* of Sikkim state (Tamang et al., 1988).

**Table 1:** Sequence statistics, alpha diversity matrix and species level taxonomy number observed for starter culture samples.

Samples	*Chowan*	*Dawdim*	*Hamei*	*Humao*	*Khekhrii*	*Phut*
Sequences used for analysis	292996	292996	292996	292996	292996	292996
**Alpha Diversity Indices**					
Observed OTUs	836	750	3037	702	1122	692
Goods coverage	0.997812	0.998276	0.990399	0.998198	0.996997	0.998601
Shannon	2.33745	3.489771	2.566343	2.645813	2.473556	2.075269
Simpson	0.689639	0.837917	0.759854	0.771975	0.684789	0.498496
Chao1	5200.255	3457.66	47476.08	3726.522	6894.537	2066.508
**Taxonomy based analysis:**					
Total number of observed species	82	108	67	82	113	142
Number of rare-species	31	39	30	24	54	59


**FIGURE 3:**
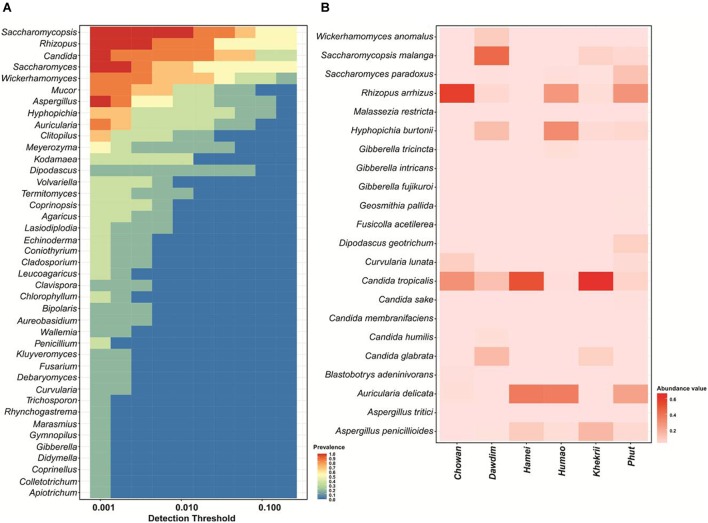
Heatmap of OTUs showing **(A)** Core genus distribution pattern and **(B)** Core species and their relative abundance present in all samples.

We found 45 core OTUs which were universally present in all starter samples tested and were further weighed to 41 genera level (Figure 3A) and 22 species level taxonomy (Figure 3B). A wide diversity of fungal species, as well as various unique species in samples has been observed in this study. A total number of 594 fungal species were detected by HTS including noble or unique species (133), common species (224) and rare-species (237), in samples of *chowan, dawdim, hamei*, *humao, khekhrii*, and *phut* (Supplementary Table 1). A total of 133 fungal species were found to be noble or unique species with reference to diversity compared to the common species, out of which 40 species were sample-specific in *phu,t* followed by *khekhrii* (28), *hamei* (23), *dawdim* (21), *chowan* (13), and *humao* (8), respectively (Supplementary Table 1). Dominant unique species based on abundance were *Tetracladium setigerum* in *khekhrii*, *Saccharomyces eubayanus* in *chowan*, *Solicoccozyma terrea* in *hamei*, *Penicillium sumatraense* in *phut*, *Acremonium implicatum* in *humao*, and *Thermomyces lanuginosus* in *hamei*. Species with less than 1% abundances are known as rare-phylotypes (Li et al., 2018). We found 237 species within the rare-phylotypes category [those with less than 1% abundances (Supplementary Table 1)] including 19 different class level taxa (Supplementary Figure 1). Interestingly, samples of *phut* had the highest number of 59 rare-species, followed by *khekhrii* (54), *dawdim* (39), *chowan* (31), *hamai* (30), and *humao* (24), respectively (Table 1). A phylotype, often referred to as OTUs, is an environmental DNA fragment or group of sequences sharing greater than 97–98% similarity of a particular gene marker (Bhadury et al., 2011; Rivett and Bell, 2018). Importantly, in such lesser-known traditionally prepared dry starters, the presence of sizable number of rare-phylotypes may have some functional or biochemical properties, and sometimes these rare-species may have human health perspectives (Bhute et al., 2017).

The unique mold species recorded in dry starters of North East India are *Aspergillus penicillioides, Rhizopus arrhizus, Rhizopus microsporus* and the unique yeast species are *Kluyveromyce smarxianus, Trichomona scusciferrii, Candida humilis, Candida metapsilosis, Saccharomyces paradoxus, Saccharomycopsis malanga*, and *Wickerhamomyces sydowiorum*. Earlier reports demonstrated the presence of common yeasts in most of the Asian dried starters, which were similar to starters of North East India, including *Candida glabrata, Cryptococcus heveanensis, Cry. albidus, Pichia fabianii, P. guilliermondii, Rhodosporidium toruloides*, *Rhodotorula mucilaginosa, Saccharomyces cerevisiae, Saccharomycopsis fibuligera, Saccharomycopsis malanga, Sporobolomyces nylandii*, and *Wickerhamomyces anomalus* (Tsuyoshi et al., 2005; Xie et al., 2007; Jeyaram et al., 2008; Thanh et al., 2008; Lv et al., 2013; Bora et al., 2016; Sha et al., 2016, 2017, 2018). We assume that the higher yeast diversity in our study could have resulted from a larger sampling population. The yeast *Saccharomycopsis fibuligera*, possessing amylase and ethanol producing capacity, is one of the most common yeasts present in dried starters of Asia (Hesseltine and Kurtzman, 1990; Limtong et al., 2002; Tsuyoshi et al., 2005; Thanh et al., 2008; Sha et al., 2018).

We correlated mycobiome diversity which was earlier detected in six samples of dry starters of India viz. *(chowan, dawdim, hamei*, *humao, khekhrii*, and *phut*) by the culture-dependent method (ITS-PCR) (6 species) and culture-independent method using PCR-DGGE analysis (24 species) (Sha et al., 2018), with that of 594 fungal species detected by high-throughput sequencing method (Supplementary Figure 2). Based on OTUs, the HTS method could detect 594 fungal species showing a diverse profile of mycobiome communities in the six different types of starters in this study, which were not earlier detected by ITS-PCR and PCR-DGGE methods (Sha et al., 2018). The read length required by HTS platforms for DNA metabarcoding is preferably 200–400 bp (Banchi et al., 2018), which is used for ITS2 gene amplification that can generate the amplicons up to 400bp in size necessary for library preparation on Illumina platform (Blaalid et al., 2013). The shorter sequences for HTS platform using ITS2 primers favor the identification of a wide range of fungi, which is a major advantage of the ITS2 primer (Bellemain et al., 2013). Whereas in ITS-PCR, the read length of ITS gene sequence amplified by primers, ITS1 and ITS4, is ranging from 600 to 750 bp (White et al., 1990), which may not be used for the library preparation in Illumina sequencing platform for HTS (Banchi et al., 2018). Amplicon-based high-throughput sequencing reveals comprehensive microbial communities with superior sequence coverage and inter- and intra-species diversity within a particular genus or among genera (Bokulich and Mills, 2013; Yan et al., 2013; Połka et al., 2015), comparable to other molecular tools. This is because HTS can generate far more reads than traditional culture-independent methods such as PCR-DGGE and facilitates the discovery of more microbiota diversity (Ercolini, 2013). However, a combined (culture-dependent and culture-independent) approach can be an appropriate strategy to investigate entire microbial communities of any food sample.

We assume that the geographical environment (including altitudes and climate) play important roles, over the production methods of dried starter cultures, when influencing the composition of microbiota (Jeyaram et al., 2011; Nam et al., 2012; Lv et al., 2012, 2013). Besides these, other factors that may affect the composition of mycobiome communities in dried starters include the level of hygienic conditions, quality of the glutinous rice or other cereal substrates, quality and source of water, as well as the back-slopping techniques used by the ethnic people (Capozzi and Spano, 2011; Gonelimali et al., 2018; Sha et al., 2018). There may also be the possibility of air-borne resources of fungal diversity in these tested samples (Cuadros-Orellana et al., 2013; Aguayo et al., 2018), probably during traditional preparation of starter.

**FIGURE 4:**
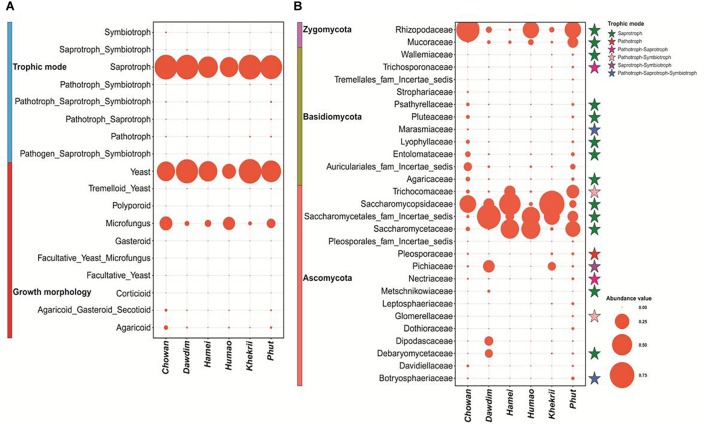
**(A)** Bubble plot describing the features of mycobiome with relative abundance distribution present in starters. **(B)** Bubble plot describing the relative abundance distribution of family level diversity and their trophic mode of nutrition found in dried starters. Family level diversity having >1.0% abundance in each sample was taken for this plot.

**FIGURE 5:**
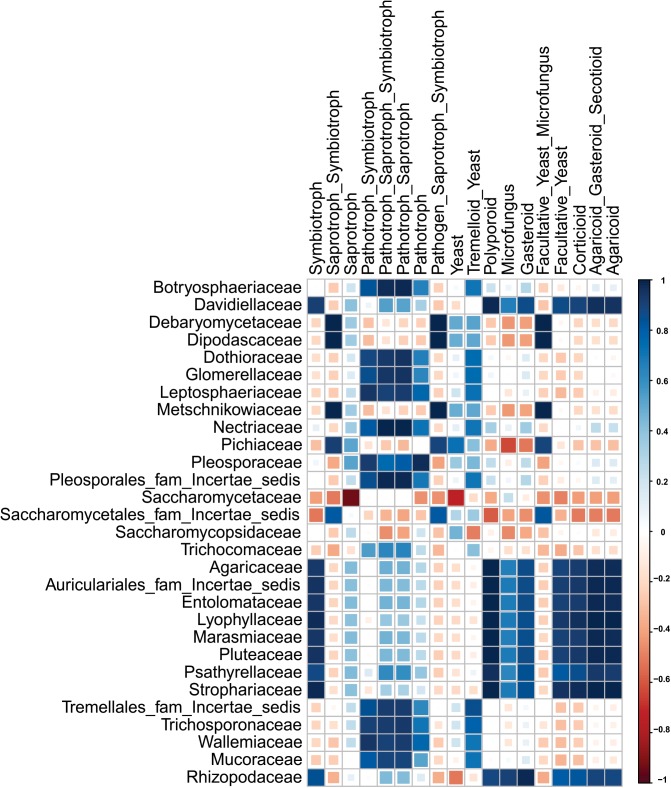
Plot showing the spearman correlation values for family level diversity having >1.0% abundance in each sample and different features of mycobiome.

The percentage distribution of total yeast and mold species found in different starters with their respective morphology and mode nutrition is shown in Supplementary Table 2. Saprotrophic mode of mold species was encountered in starters with a dominance range of 64 to 99% over other modes. In other hands, yeast morphotypes were more dominants in all samples (Figure 4A). Several families were enlisted for the diversity players inside the starters; most of them were saprophytes irrespective of the taxonomy. The Saccharomycopsidaceae family showing the saprotrophic mode of nutrition were found to be abundant, and the pathogenic Pleosporaceae family (Ariyawansa et al., 2015) had a lower abundance in Ascomycota phylum (Figure 4B). Most of the families were associated with the functional attributes to the KEGG Orthologous for the eubacterial diversity. Some important correlations have been observed between families and functional guilds (Figure 5). Interestingly, Pichaceae was negatively correlated to the micro-fungus morphotypes, and such correlations have been suggesting the extrusion of the diversity simulation (Schoch et al., 2012).

Functional attributes of the fungal diversity were formulated with bioinformatics tools, based on methods described by Gweon et al. (2015) and Nguyen et al. (2016). Since the ITS region has been recognized as the universal barcode for identification of fungi (Schoch et al., 2012), we used this region for fungal bar code with reference to database UNITE for OTU assignment. We applied the PIPITS pipeline since it extracts the ITS sub-region from raw reads and assigns taxonomy with a trained RDP Classifier. Total 662461 sequences were identified out of 689459 sequences, as containing an ITS2 sub-region. After quality filtering and removal of contaminants, we obtained results in 2402833 quality sequences. We set 59612 sequences per sample for further analysis to form 354 OTUs, which yielded 190 phylotypes. Several functions of the mycobiome were observed after the funguild function analysis (Supplementary Table 3). However, comparing with culture-independent method, the culturable diversity is more relevant for development of a potent starter in beverage industries (Sha et al., 2018).

## Conclusion

Our study has shown a wide diversity of yeast and mold species (594 fungal species) in dry starters of North East India, based on nucleotides sequences clustered into OTUs, following the amplicon sequencing using a high-through sequence platform as well as bioinformatics tools. Taxonomical identifications of some sample-specific species of mycobiome in these starters are a remarkable observation in lesser-known, traditionally prepared dry starters for alcohol production in India. The present study demonstrated the baseline data for mycobiome diversity in traditionally prepared dry starters of India.

## Author Contributions

SS conducted the major experiments. MS has assisted with the bioinformatics. JT supervised the experiments and finalized the manuscript.

## Conflict of Interest Statement

The authors declare that the research was conducted in the absence of any commercial or financial relationships that could be construed as a potential conflict of interest.

## Footnotes

1 www.northeasttourism.gov.in

2 http://www.bioinformatics.babraham.ac.uk/projects/fastqc/

3 https://github.com/torognes/vsearch

4 http://www.stbates.org/guilds/app.php

5 https://www.r-project.org/

## Supplementary Material

Figure S1 Distribution of rare-phylotypes category with 19 different class level taxa in six starters of North East India.

Figure S2 Correlation among mycobiome diversity of six starters (*chowan, dawdim, hamei*, *humao, khekhrii*, and *phut*) **A**: 6 species of yeasts by culture-dependent method (ITS-PCR) (Sha et al., 2018), **B**: 24 species (yeasts = 6 and molds = 24) by PCR-DGGE analysis (Sha et al., 2018) and **C**: Total 594 species (yeasts = 83 and molds = 511) by high-throughput sequencing technique. Number and identity of each species are presented in Supplementary Table 1.

Table S1 Table showing total number of 594 fungal species detected by HTS including common species (224), unique species (133) and rare-species (237), and their taxonomic affiliations in samples of *chowan, dawdim, hamei*, *humao, khekhrii*, and *phut*. Table also shows number of yeast species (6) detected by ITS-PCR, and yeast (18) and mold species (6) by PCR-DGGE in the same samples as reported earlier by Sha et al. (2018).

Table S2 Table showing percent distribution of total yeast and mold species in different starters with their respective morphology and mode nutrition.

Table S3 Different operational taxonomic units (OTUs) with taxonomy and their respective functional attributes observed in different dried starters. Generation of OTUs and functional attributes were derived through PIPITS (Gweon et al., 2015) and FUNGuild (Nguyen et al., 2016) environments.
