# Supplementary figures and images for: Mycobiome Diversity in Traditionally Prepared Starters for Alcoholic Beverages in India by High-Throughput Sequencing Method
Source: Front Microbiol. 2019 Mar 5;10:348. doi: 10.3389/fmicb.2019.00348 (PMC6411702; doi:10.3389/fmicb.2019.00348)

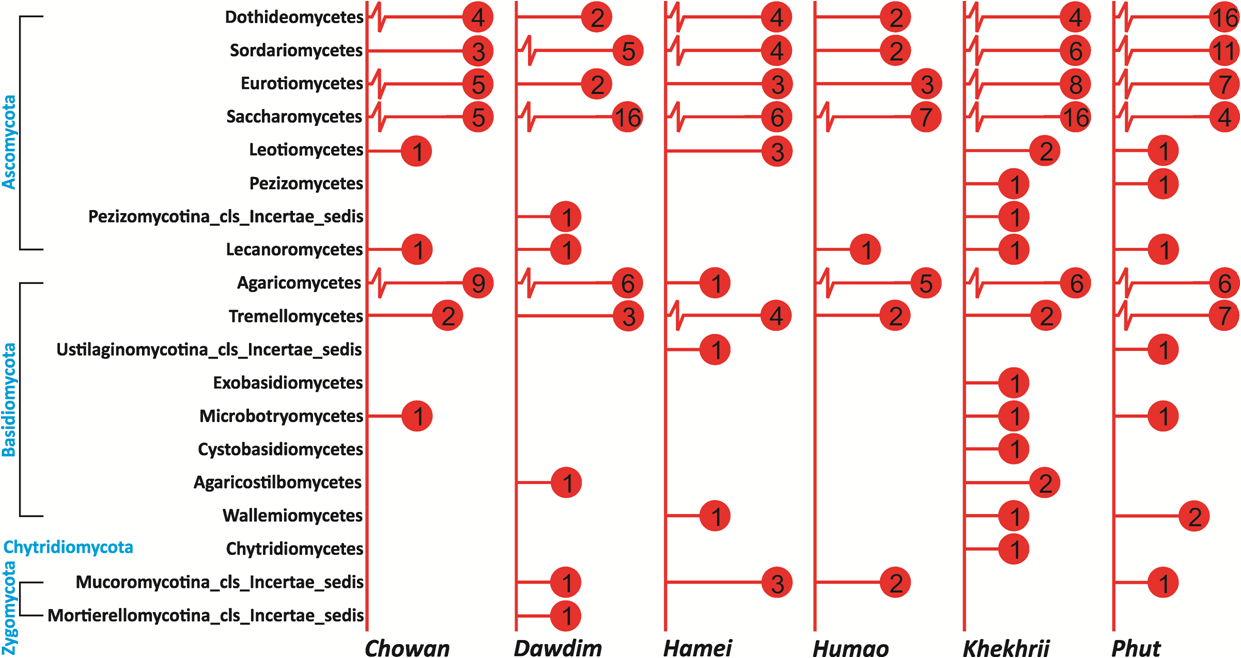

Supplement: Figure S1 — Distribution of rare-phylotypes category with 19 different class level taxa in six starters of North East India. [file Image_1.TIF]

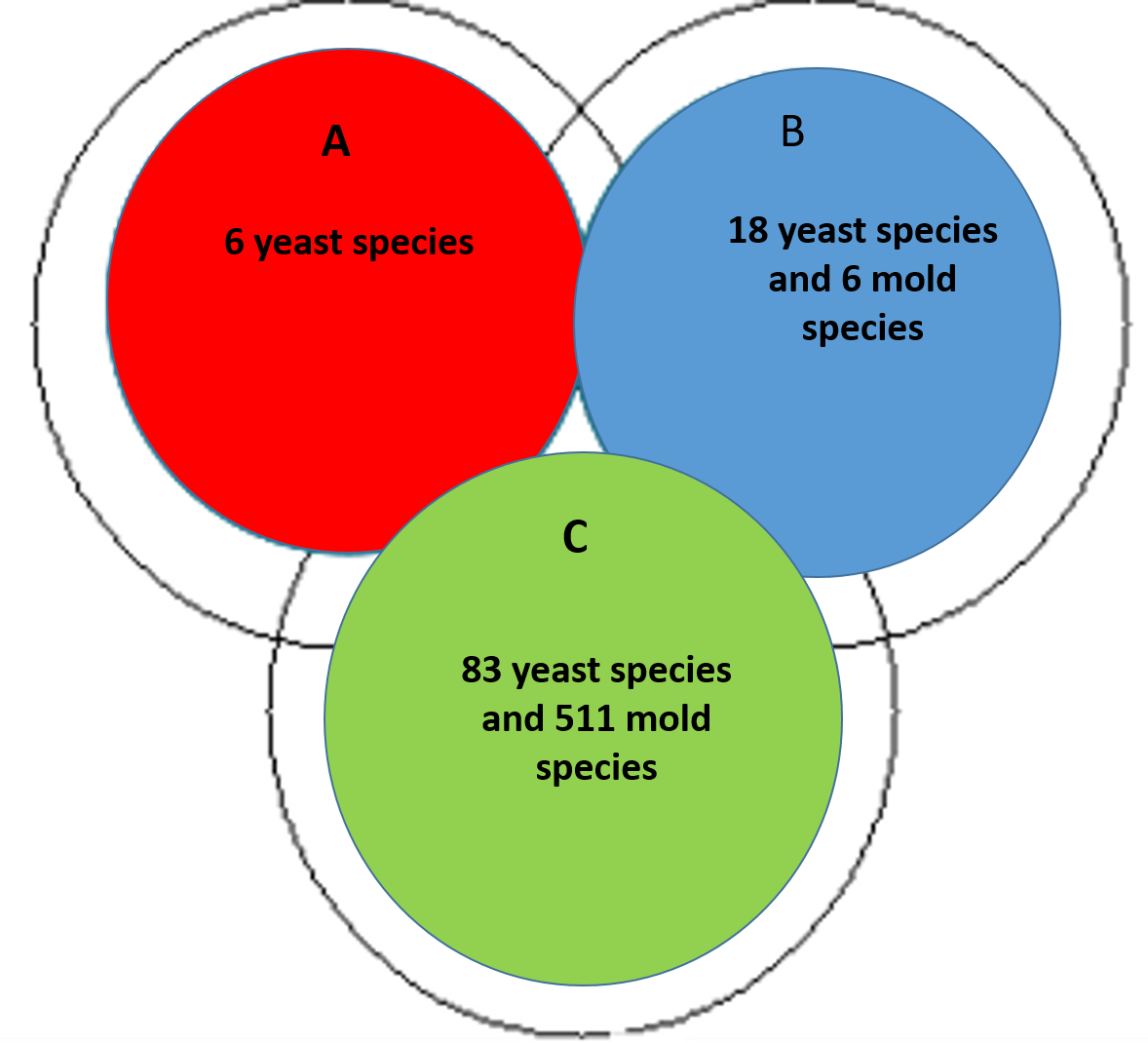

Supplement: Figure S2 — Correlation among mycobiome diversity of six starters (chowan, dawdim, hamei, humao, khekhrii, and phut) A: 6 species of yeasts by culture-dependent method (ITS-PCR) (Sha et al., 2018), B: 24 species (yeasts = 6 and molds = 24) by PCR-DGGE analysis (Sha et al., 2018) and C: Total 594 species (yeasts = 83 and molds = 511) by high-throughput sequencing technique. Number and identity of each species are presented in Supplementary Table 1. [file Image_2.TIFF]
